# Clinical Characteristics Associated with the Development of Cystoid Macular Edema in Patients with Cytomegalovirus Retinitis

**DOI:** 10.3390/microorganisms9061114

**Published:** 2021-05-21

**Authors:** Hye-Ji Kwon, Gisung Son, Joo-Yong Lee, June-Gone Kim, Yoon-Jeon Kim

**Affiliations:** 1 Department of Ophthalmology, Asan Medical Center, University of Ulsan College of Medicine, Seoul 05505, Korea; hyejigracekwon@gmail.com (H.-J.K.); ophthalmo@amc.seoul.kr (J.-Y.L.); junekim@amc.seoul.kr (J.-G.K.); 2 Department of Retinal Service, Hangil Eye Hospital, Incheon 21388, Korea; yatase@hanmail.net

**Keywords:** cytomegalovirus retinitis, cystoid macular edema, risk factors, visual prognosis

## Abstract

We evaluated the incidence and characteristics of eyes with cytomegalovirus (CMV) retinitis according to the occurrence of cystoid macular edema (CME) and identified the risk factors of its occurrence. Patients diagnosed with CMV retinitis and examined using optical coherence tomography were classified according to the development of CME. The CME group was further divided according to the presence of active retinitis at the time of CME development. The demographics, serologic findings, ophthalmic presentations, ocular treatments, and visual prognosis were compared. CME was identified in 25 eyes (17 eyes with active retinitis and 8 eyes with inactive retinitis) out of the 67 eyes with CMV retinitis. Visual acuity was worse in the CME group than in the non-CME group. The CME group had longer CMV viremia duration, zone 1 involvement, and larger extent of CMV retinitis. While CME with concurrent active retinitis developed in eyes with direct foveal involvement of retinitis in the acute phase and required more ganciclovir injections after CME development, CME without active retinitis developed in eyes with larger extents of involvement and more intravitreal ganciclovir injections before CME development. Zone 1 involvement and longer CMV viremia duration were independently associated with the occurrence of CME. CME, which caused visual deterioration, developed in considerable patients with CMV retinitis and had different characteristics according to the presence of active retinitis.

## 1. Introduction

Cytomegalovirus (CMV) is a ubiquitous herpes family virus with a prevalence of approximately 50% in the latent form in the general population [1,2]. CMV retinitis is one of the most common opportunistic ocular infection in immunocompromised patients [3,4]. After the advent of antiretroviral therapy (ART), the incidence of CMV retinitis in patients infected with human immunodeficiency virus (HIV) declined and their visual outcomes improved [3]. On the other hand, the incidence of CMV retinitis has been increasing in the past 15 years in non-HIV patients with diseases such as hematopoietic malignancy, solid organ transplantation, or autoimmune diseases [1,2,3,4,5].

Cystoid macular edema (CME) is one of the causes of severe central visual loss in CMV retinitis [6,7]. CME is theorized to develop as a result of intraocular inflammation in response to the CMV infection [8]. The development of optical coherence tomography (OCT) has improved the evaluation of CME, and CME associated with CMV retinitis has been reported from small case series [9,10]. The purpose of this study was to describe the clinical characteristics of patients with eyes with CMV retinitis according to the presence of CME, and to identify the factors associated with CME occurrence in eyes with CMV retinitis.

## 2. Materials and Methods

### 2.1. Study Subjects

**Figure 1:**
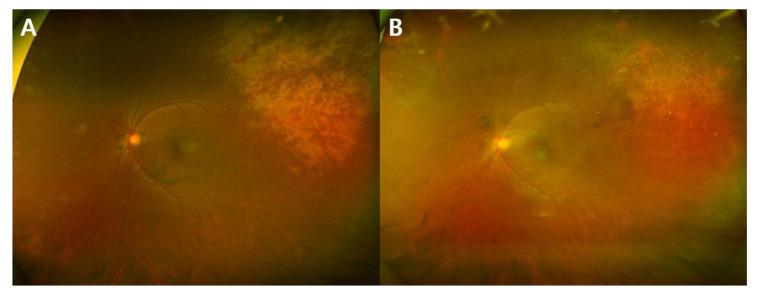
Representative fundus photos of the same patient showing the active and inactive stages of cytomegalovirus (CMV) retinitis: (**A**) active retinitis with infiltration; and (**B**) inactive retinitis with resolution of infiltration.

We identified patients with CMV viremia who were referred to the ophthalmology department at Asan Medical Center (Seoul, Korea) for the screening of CMV retinitis between March 2008 and February 2018. Among them, we included those who were diagnosed with CMV retinitis with follow-up durations of longer than 3 months and reviewed their medical records in a consecutive manner. Among them, those who received OCT exams more than two times were included in this retrospective observational cohort study. Patients were excluded if OCT images were not of sufficient quality to be interpreted due to media opacity or poor patient cooperation. Patients with retinal vascular disease such as retinal vein occlusion or diabetic retinopathy which could potentially cause macular edema were also excluded. Patients were categorized into two groups according to the development of CME: CME group vs. non-CME group. The presence of CME was defined when the hyporeflective intraraetinal cystic space was observed in three consecutive OCT scans. CME group were further investigated based on presence of active retinitis at the time of CME development. The representative fundus images of eyes with and without active retinitis are shown in Figure 1. The study was approved by the Institutional Review Board of Asan Medical Center before its initiation (IRB No. 2019-1457), and the study was carried out in compliance with the tenets of the Declaration of Helsinki. The institutional review board of Asan Medical Center waived the need for written informed consent considering the retrospective nature of the study and the use of deidentified patient data.

### 2.2. Ocular Exams and Treatments

The fundus of every patient referred to the ophthalmology department was thoroughly examined by retinal specialists by steering fundus photograph, ultra-widefield fundus photography (Optos 200Tx system, Optos plc, Dunfermline, United Kingdom), or both. The OCT images were taken using the Spectralis (Heidelberg Engineering) to assess the presence of macular abnormalities. The time interval between the OCT scans were as follows: 2–3 weeks in CME eyes regardless of the presence of active retinitis; 1–2 months in non-CME eyes with active retinitis; and 6 months in non-CME eyes without active retinitis. The diagnosis of CMV retinitis was based on clinical features and CMV viremia. As soon as the patients were diagnosed with CMV retinitis, systemic ganciclovir treatment was started with the induction dose (5 mg/kg IV every 12 h) for 14–21 days and then maintained in the maintenance dose (5 mg/kg IV every 24 h). Systemic foscarnet (60 mg/kg IV every 8 h or 90 mg/kg every 12 h) was used in cases in which systemic ganciclovir treatment could not be used because of severe neutropenia or ganciclovir-resistant CMV infection. Antiviral prophylaxis of systemic ganciclovir (5 mg/kg IV every 24 h) was administered before the diagnosis of CMV disease in patients with high risks according to the routine protocols of respective departments. Besides systemic antiviral therapy, intravitreal ganciclovir injection (2 mg/0.1 mL) was administered in patients who did not improve after systemic treatment or had infiltration in zone 1. The number and interval of ganciclovir injections were individualized according to the size and location of the lesion and treatment response. Vitrectomy was performed when retinal detachment or non-clearing vitreous hemorrhage occurred during follow-up.

### 2.3. Outcome Measures

**Figure 2:**
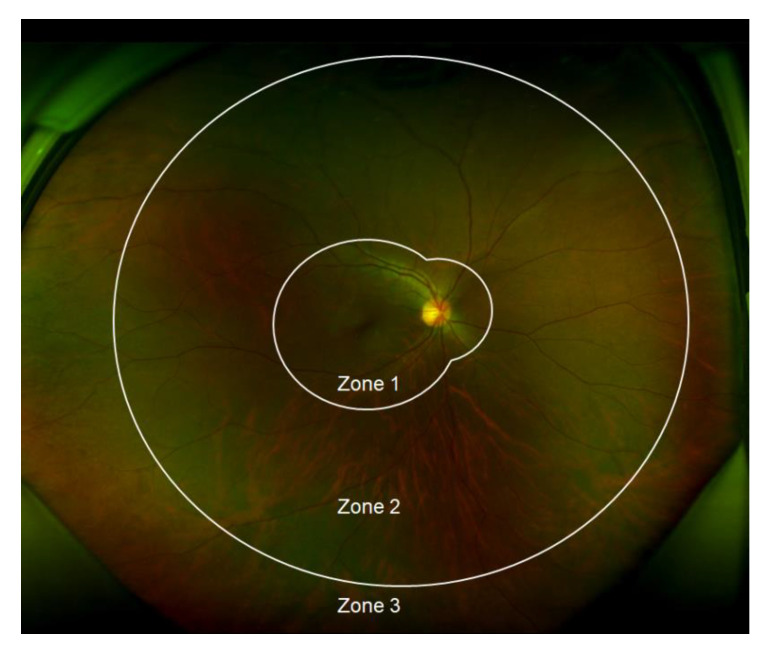
Zones involved of cytomegalovirus (CMV) retinitis. Zone 1 is defined as the area within 1500 µm of the optic nerve or 3000 µm of the fovea. Zone 2 includes the area outside from Zone 1 to the equator (as defined by the vortex veins). Zone 3 includes the peripheral retina anterior to the equator extending to the ora serrata.

The following variables were examined in each patient: (i) demographic variables (i.e., age, sex, underlying disease, total follow-up period, and mortality); (ii) clinical laboratory variables (i.e., peak CMV DNA polymerase chain reaction (PCR) titer [5], absolute neutrophil count (ANC) at diagnosis, and duration of CMV viremia); and (iii) ocular variables (i.e., best-corrected visual acuity (BCVA), presence of active retinitis at the time of CME development, the extent of disease involvement, number of intravitreal ganciclovir injections, recurrence of CMV retinitis, and ocular complications). As for BCVA, measurements at the initial visit and the last follow-up visit were used. The involved zones of CMV retinitis were categorized as zones 1, 2, and 3 according to the area of involved fundus (Figure 2). Responses to treatment were categorized as complete resolution, partial resolution, and no response. Cases with complete disappearance of CME after 3 months of treatment were categorized into the complete resolution group, and cases in which CME remained or relapsed within 3 months after discontinuation of treatment despite short-term improvement were categorized into the incomplete resolution group. Cases showing no improvement after 3 months of consecutive treatment were categorized into the no response group.

### 2.4. Statistical Analysis

Descriptive statistics (number and percentage of each categorical variable and the median and interquartile range of each continuous variable) were analyzed to characterize the baseline characteristics of the subjects. The Shapiro–Wilk test was used to explore the distribution of numerical data. To demonstrate the characteristics of subjects with CME development in CMV retinitis compared with those without, the Mann–Whitney U test was used for non-normally distributed numerical data. To compare categorical data, Fisher’s exact test was used. Risk factors for CME development were calculated as well. Odds ratios (ORs) for associations among potential risk factors were obtained using the generalized estimating equation to manage the non-independency of eyes in the same patient in which the development of CME was the dependent variable. Univariate analyses were performed separately for each variable, and variables with probability values less than 0.1 in univariate analysis were included in the multivariable regression analysis model. The ORs with 95% confidence intervals (CIs) were calculated. All statistical analyses were performed using the SAS software, version 9.4 (SAS Institute Inc., Cary, NC, USA).

## 3. Results

Among 570 patients who were screened at our center, 114 eyes of 83 patients were diagnosed with CMV retinitis. After excluding those with OCT images of poor qualities, 67 eyes of 48 patients (38 male and 10 female) were finally included in the analysis. The median age of subjects was 51.5 years. The most common underlying disease was hematologic malignancy (27 patients, 56.2%), followed by solid organ transplantation (14 patients, 29.2%) and HIV infection (6 patients, 12.5%). PCR testing using aqueous humor was conducted in subset of patients (32 eyes among 67 eyes). As a result, the positive rate of CMV PCR in aqueous humor was same as 75% in both groups: CME group (12 eyes from 16 eyes) and non-CME group (12 eyes from 16 eyes).

### 3.1. Incidence and Clinical Characteristics of CME in CMV Retinitis

During the median 20.37 months of follow-up, CME developed in 25 (37%) eyes of 23 patients at median 1.40 months after the initial diagnosis of CMV retinitis. Table 1 shows the clinical characteristics of CME in CMV retinitis. Patients with CME were older than those without CME (*p* = 0036). No significant differences were observed in other demographic variables such as sex, underlying disease, and total follow-up period according to the development of CME. The CME group had longer viremia period (CME: median 2.57 months vs. non-CME: median 2.10 months, *p* = 0.024). Zone 1 involvement (80.0% vs. 35.7%, *p* = 0.001) and ≥ 50% extent of total retina (64.0% vs. 19.0%, *p* < 0.001) involvement were more frequently observed in the CME group. The total numbers of intravitreal ganciclovir injections (*p* < 0.010) and eyes that received intravitreal treatment (*p* = 0.013) were higher in the CME group. Baseline logMAR BCVA did not significantly differ according to the presence of CME (*p* = 0.350); however, the final BCVA was significantly worse in the CME group (*p* < 0.001), which showed significant visual deterioration during follow-up (*p* = 0.001). There was no significant difference in the lens status between the two groups.

### 3.2. Clinical Characteristics of CME Eyes in CMV Retinitis according to the Presence of Active Retinitis

**Figure 3:**
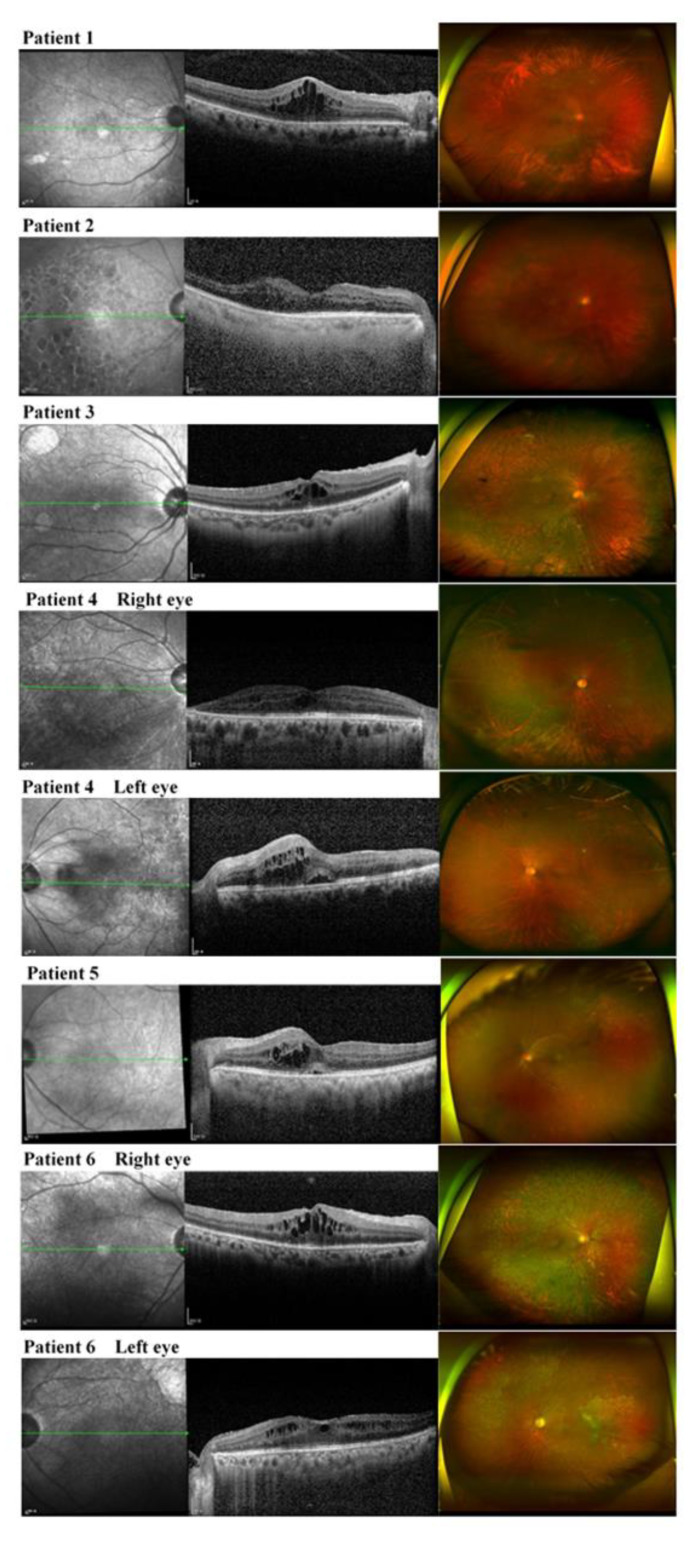
Widefield fundus photos and optical coherence tomography images of patients with cystoid macular edema (CME) development in cytomegalovirus (CMV) retinitis without active retinitis (eight eyes of six patients). CME in inactive CMV retinitis developed in cases with longer viremia, higher prevalence of recurrence rate, larger extent of retinal involvement, and these patients showed treatment responses to topical nonsteroid anti-inflammatory drugs (NSAIDs).

CME eyes had different characteristics according to the presence of active retinitis at the time of CME development. Therefore, the CME group was further divided into two groups according to the presence of active retinitis: CME with concurrent active retinitis (17 eyes of 17 patients) vs. CME without concurrent active retinitis (8 eyes of 6 patients, Figure 3). Whereas CME with concurrent active retinitis developed in the earlier phase (median 0.17 months after the initial diagnosis of CMV retinitis), CME without active retinitis developed in the later phase (median 15.80 months after the initial diagnosis of CMV retinitis, *p* < 0.001). No significant differences were noted between the two groups regarding age, sex, and underlying disease (Table 2).

CME without active retinitis had longer viremia (median 16.75 months vs. median 1.80 months, *p* = 0.050) and showed a higher prevalence of recurrence (87.5% vs. 11.8%, *p* = 0.001), higher rate of posterior vitreous cell (100.0% vs. 70.6%, *p* = 0.140), and higher frequency of involvement of the larger extent of the total retina ≥ 50% (100% vs. 47.1%, *p* = 0.022). As for the number of intravitreal ganciclovir injections, the total number was not significantly different between two groups, but the treatment number was different before and after the development of CME: eyes without active retinitis had a larger number of intravitreal ganciclovir injections before the development of CME (median 13.00 vs. median 0.00, *p* = 0.006), whereas eyes with active retinitis had more injections after the development of CME (median 1.00 vs. median 8.00, *p* = 0.059). Neither baseline nor final BCVA significantly differed between the two groups.

Treatment modalities also significantly differed according to the presence of active retinitis (Table 3). Particularly, systemic antiviral treatments differed according to the presence of active retinitis (*p* < 0.001). Intravitreal ganciclovir injection was administered in 14 (82.4%) eyes among those with active retinitis and in only 2 (25.0%) eyes among those without active retinitis (*p* = 0.010). In total, six eyes received intravitreal bevacizumab alone or in combination with ganciclovir; in the rest of the cases, topical nonsteroidal anti-inflammatory drugs (NSAIDs) or topical steroid agents were used. Whereas 11 (64.7%) eyes with active retinitis showed resolution of CME after intravitreal antiviral treatment, only 2 (25.0%) eyes without active retinitis showed resolution of CME after topical treatment with NSAIDs.

### 3.3. Risk Factors for the Development of CME in CMV Retinitis

In a multivariable regression analysis to identify the clinical factors associated with CME occurrence in eyes with CMV retinitis (Table 4), zone 1 involvement of retinitis was the most strongly associated with CME development (OR = 4.25; *p* = 0.021). In addition, longer CMV viremia duration (OR = 1.10; *p* = 0.045) was found to be independently associated with the development of CME in patients with CMV retinitis. Larger extent involvement of CMV retinitis (OR = 3.82; *p* = 0.062) was associated with CME development with borderline statistical significance.

## 4. Discussion

In our study, a significant portion of eyes with CMV retinitis (37.3%) developed CME, particularly in eyes with longer duration of CMV viremia and advanced type of CMV retinitis with extensive retinal involvement. Eyes with CME showed significant deterioration of visual acuities. To the best of our knowledge, this is the first study to longitudinally analyze the comprehensive aspects of CME using OCT in a relatively large cohort of CMV retinitis subjects.

The pathogenesis of CME occurrence in CMV retinitis is yet to be fully delineated. Our data show that the clinical features and backgrounds of CME are different depending on the timing of CME development and the presence of active retinitis at the time of CME occurrence; in addition, eyes with direct foveal involvement of CMV infiltration developed CME in the early phase. Gupta et al. [10] reported that CME was observed in 20% of patients with CMV retinitis with macular involvement; because of their central involvement, these patients were treated with intravitreal ganciclovir injections after the development of CME.

Compared with CME eyes with active retinitis, we found that those without active retinitis had larger retinal involvement, longer viremia duration, and higher recurrence rate. These characteristics are similar to those with immune recovery uveitis, an inflammatory syndrome observed in HIV patients with CMV retinitis receiving ART. In our study, only 12.5% of the patients were HIV-positive; therefore, further studies are needed to determine why a large portion of HIV-negative patients with CMV retinitis show overlapping characteristics with HIV-positive patients. Previous studies suggested that the development of CME is associated with inflammation due to the restoration of immune competence. Weinberg et al. [11] and Karavellas et al. [12] described CME cases of inactive CMV retinitis in HIV patients, which were attributed to chronic intraocular inflammation and recovered immunity. In our study, whereas antiviral treatments were effective in CME patients with active retinitis, anti-inflammatory treatments with topical NSAIDs were effective in CME patients without infiltration. The pathogenesis of CME is considered to be different between these two subsets described. The eyes with active retinitis have active infectious inflammation that causes CME, and the eyes with inactive retinitis presumably have post infectious inflammation driven by residual viral antigen in the retina and vitreous. Taken together, it is plausible that the aforementioned risk factors associated with CME development in eyes with inactive CMV retinitis induce more viral antigens in the retina, and the subsequent reactive inflammation might be the main cause of CME development.

We also observed that CME eyes of patients with inactive CMV retinitis had a history of a higher number of intravitreal ganciclovir injections. It is not clear whether intravitreal ganciclovir injection could be a risk factor for the development of CME. Repeated injections of ganciclovir do not appear to be associated with a high risk of complications; however, in a rabbit model, multiple intravitreal injections of ganciclovir resulted in vacuolization of photoreceptor inner segments [13]. The other possibility is that variables that necessitate more intravitreal ganciclovir injections (e.g., larger extent of retinal involvement, higher viral load, and longer CMV viremia duration) may themselves act as risk factors of CME development in CMV retinitis.

Wong et al. [14] suggested that the lens status may be a contributing factor to CMV uveitis-associated CME, which was support by another report that the protection of lens against CME is weakened after cataract surgery [15]. However, in our study, there was no significant difference in CME occurrence according to the lens status.

This study has several limitations. Because of the nature of its retrospective design, the number and intervals of OCT were not uniform across patients. In addition, the patients were treated by four different retina specialists at our center; as such, the treatment plans for CME slightly varied among the retina specialists and hindered a rigorous analysis for treatment efficacy. Lastly, we performed the diagnostic approaches using ocular fluid directly in subset of patients. To reduce the possible errors in clinical diagnosis of CMV retinitis, however, we strictly included only cases with characteristic features of CMV retinitis in this study.

## 5. Conclusions

The incidence of CME was quite high among eyes with CMV retinitis and was closely related to decreased visual acuity. CME was more likely to occur in patients with CMV retinitis with zone 1 involvement and longer duration of CMV viremia. To minimize the deterioration of visual function due to CME in at-risk patients with CMV retinitis, serial ophthalmologic exams with OCT and timely ocular management will be helpful.

## Figures and Tables

**Table 1:** Demographics and clinical characteristics of eyes with cytomegalovirus (CMV) retinitis according to the development of cystoid macular edema (CME).

Patient Level				
	Total (48 patients)	No CME (25 patients)	CME (23 patients)	*p* Value
**Demographics**				
Age, years	51.50 [35.00, 58.25]	45.00 [31.00, 54.00]	57.00 [46.50, 61.00]	0.036
Female, N (%)	10 (20.8)	7 (28.0)	3 (13.0)	0.292
**Underlying disease, N (%)**				0.304
HIV	6 (12.5)	2 (8.0)	4 (17.4)	
Solid organ transplantation	14 (29.2)	6 (24.0)	8 (34.8)	
Hematologic malignancy	27 (56.2)	17 (68.0)	10 (43.5)	
Other disease	1 (2.1)	0 (0.0)	1 (4.3)	
Mortality during follow-up, N (%)	13 (27.1)	8 (32.0)	5 (21.7)	0.523
Total follow-up period, months	20.37 [3.30, 40.16]	14.37 [2.23, 45.10]	22.40 [8.03, 34.63]	0.445
**Clinical Laboratory Methods**				
Peak CMV PCR level, log	3.92 [3.15, 5.13]	3.87 [3.19, 4.83]	3.97 [2.91, 5.45]	0.725
ANC at diagnosis, count	2048.50 [990.00, 3582.50]	1624.00 [930.00, 2500.00]	2140.00 [1050.00, 4060.00]	0.317
Viremia period, months	2.18 [1.16, 5.15]	2.10 [0.80, 3.27]	2.57 [1.27, 12.78]	0.024
**Ocular Characteristics**				
Bilateral involvement, N (%)	19 (39.6)	11 (44.0)	8 (34.8)	0.566
**Eye Level**				
	**Total** **(67 eyes)**	**No CME** **(42 eyes)**	**CME** **(25 eyes)**	***p* value**
**Ocular Characteristics**				
Lens status: Pseudophakia, N (%)	7 (10.4)	3 (7.1)	4 (16.0)	0.411
Involvement extent, N (%)				
Zone 1 involvement	35 (52.2)	15 (35.7)	20 (80.0)	0.001
Foveal involvement	19 (28.4)	10 (23.8)	9 (36.0)	0.401
≥ 50% involvement of total retina	24 (35.8)	8 (19.0)	16 (64.0)	< 0.001
Intravitreal GCV injection				
Total number of injections, N	5.00 [0.00, 10.00]	3.00 [0.00, 6.75]	10.00 [5.00, 17.00]	< 0.001
Number of injections before CME, N	2.00 [0.00, 8.50]	3.00 [0.00, 6.75]	0.00 [0.00, 11.00]	0.798
Eyes with injections, N (%)	46 (68.7)	24 (57.1)	22 (88.0)	0.013
Recurrence of CMV retinitis, N (%)	18 (26.9)	9 (21.4)	9 (36.0)	0.256
Vitrectomy, N (%)	15 (22.4)	7 (16.7)	8 (32.0)	0.225
Visual outcome, LogMAR				
Baseline VA	0.20 [0.10, 0.30]	0.20 [0.10, 0.40]	0.10 [0.00, 0.30]	0.350
Final VA	0.50 [0.30, 1.35]	0.35 [0.10, 0.70]	1.30 [0.50, 1.50]	< 0.001
Change in VA	0.30 [0.00, 0.90]	0.10 [−0.08, 0.38]	0.80 [0.30, 1.30]	0.001

ANC, absolute neutrophil count; GCV, ganciclovir; HIV, human immunodeficiency virus; VA, visual acuity. Data presented as median [interquartile range].

**Table 2:** Clinical characteristics of cystoid macular edema (CME) eyes in cytomegalovirus (CMV) retinitis according to the presence of active retinitis.

Patient Level			
	With Active Retinitis (17 Patients)	Without Active Retinitis (6 Patients)	*p* Value
**Demographics**			
Age, years	57.00 [50.00, 62.00]	50.50 [34.00, 58.00]	0.248
Female, N (%)	2 (11.8)	1 (16.7)	1.000
**Underlying disease, N (%)**			0.867
HIV	3 (17.6)	1 (16.7)	
Solid organ transplantation	5 (29.4)	3 (50.0)	
Hematologic malignancy	8 (47.1)	2 (33.3)	
Other disease	1 (5.9)	0 (0.0)	
Mortality during follow-up, N (%)	4 (23.5)	1 (16.7)	1.000
Total follow-up period, months	12.03 [4.90, 34.40]	26.31 [24.11, 34.35]	0.093
**Clinical Laboratory Methods**			
Peak CMV PCR level, log	3.54 [2.80, 5.43]	4.27 [3.78, 5.34]	0.420
ANC at diagnosis, count	2140.00 [1010.00, 4100.00]	2030.00 [1210.00, 3637.50]	0.889
Viremia period, months	1.80 [1.20, 4.03]	16.75 [9.07, 20.61]	0.050
**Ocular Characteristics**			
Bilateral involvement, N (%)	5 (29.4)	3 (50.0)	0.621
**Eye Level**			
	**With active retinitis** **(17 eyes)**	**Without active retinitis** **(8 eyes)**	***p* value**
**Ocular Characteristics**			
Timing of CME development, months	0.17 [0.00,2.34]	15.80 [10.98, 20.88]	< 0.001
Lens status: Pseudophakia, N (%)	2 (11.8)	2 (25.0)	0.570
Involvement extent, N (%)			
Zone 1 involvement	13 (76.5)	7 (87.5)	1.000
Foveal involvement	9 (52.9)	0 (0.0)	0.022
≥ 50% involvement of total retina	8 (47.1)	8 (100.0)	0.022
Intravitreal GCV injection			
Total number of injections, N	9.00 [5.00, 13.00]	13.50 [9.25, 23.75]	0.280
Number of injections before CME, N	0.00 [0.00, 2.00]	13.00 [8.25, 21.00]	0.006
Posterior vitreous cell, N (%)	12 (70.6)	8 (100.0)	0.140
Vitreous opacity, N (%)	6 (35.3)	4 (50.0)	0.667
Recurrence of CMV retinitis, N (%)	2 (11.8)	7 (87.5)	0.001
Vitrectomy, N (%)	4 (23.5)	4 (50.0)	0.359
Visual outcome, LogMAR			
Baseline VA	0.30 [0.10, 0.30]	0.00 [0.00, 0.10]	0.016
Final VA	1.40 [0.90, 1.50]	0.45 [0.38, 2.22]	0.279
Change in VA	1.00 [0.50, 1.20]	0.40 [0.27, 2.15]	0.793

ANC, absolute neutrophil count; GCV, ganciclovir; HIV, human immunodeficiency virus; VA, visual acuity. Data presented as median [interquartile range] for continuous variable and number (percentage) for categorical variable.

**Table 3:** Treatment modalities for eyes with cystoid macular edema (CME) in patients with cytomegalovirus (CMV) retinitis.

	Total (25 Eyes)	With Active Retinitis (17 Eyes)				Without Active Retinitis (8 Eyes)			
		Treatment Modality	Treatment Response			Treatment Modality	Treatment Response		
			Complete Resolution	Partial Resolution	No Response		Complete Resolution	Partial Resolution	No Response
**Systemic antiviral treatment**									
Ganciclovir/Foscarnet	19	17	11	5	1	2	0	1	1
**Local treatment**									
Intravitreal ganciclovir injection	13	12	9	3	0	1	0	1	0
Intravitreal bevacizumab injection	3	1	0	1	0	2	0	1	1
Intravitreal ganciclovir and bevacizumab injection	3	2	2	0	0	1	0	1	0
Topical steroid	1	0	0	1	0	2	0	2	0
Topical NSAIDs	5	2	0	1	1	3	2	1	0

NSAIDs, nonsteroidal anti-inflammatory drugs.

**Table 4:** Factors associated with cystoid macular edema (CME) occurrence in eyes with cytomegalovirus (CMV) retinitis.

	Univariate Analysis			Multivariable Analysis		
	OR	95% CI	*p* Value	OR	95% CI	*p* Value
**Demographics**						
Age	1.03	1.00–1.07	0.132			
Female (vs. male)	0.29	0.08–1.16	0.079			
HIV (vs. non-HIV)	1.92	0.42–8.77	0.399			
Mortality during follow-up	0.81	0.24–2.79	0.736			
**Serology**						
Peak CMV PCR level	1.05	0.70–1.60	0.800			
ANC at diagnosis	1.00	1.00–1.01	0.296			
Viremia period	1.11	1.04–1.18	0.002	1.10	1.00–1.20	0.045
**Ocular characteristics**						
Involvement extent						
Zone 1 involvement(vs. no involvement)	7.16	2.32–22.16	<0.001	4.25	1.25–14.48	0.021
Foveal involvement	1.94	0.58–6.62	0.288			
≥ 50% involvement of total retina	7.94	2.38–26.57	<0.001	3.82	0.94–15.57	0.062
Intravitreal GCV injection	1.00	0.97–1.07	0.532			
Recurrence of CMV retinitis	1.88	0.58–6.18	0.300			
Visual outcome						
Baseline visual acuity	1.04	0.38–2.94	0.934			

ANC, absolute neutrophil count; GCV, ganciclovir; HIV, human immunodeficiency virus; OR, odds ratio; CI, confidence interval.

## Data Availability

Data sharing not applicable.
